# The morphometrics of autopolyploidy: insignificant differentiation among sexual–apomictic cytotypes

**DOI:** 10.1093/aobpla/plz028

**Published:** 2019-06-04

**Authors:** Karin Bigl, Juraj Paule, Christoph Dobeš

**Affiliations:** 1 Department of Pharmacognosy, Pharmacobotany, University of Vienna, Althanstrasse, Vienna, Austria; 2 Department of Botany and Molecular Evolution, Senckenberg Research Institute & Natural History Museum Frankfurt, Senckenberganlage, Frankfurt/Main, Germany

**Keywords:** AFLPs, apomixis, morphology, polyploidy, *Potentilla puberula*, reproduction, Rosaceae, sexuality

## Abstract

Polyploidization of the plant genome affects the phenotype of individuals including their morphology, i.e. size and form. In autopolyploids, we expect mainly nucleotypic effects, from a number of monoploid genomes (i.e. chromosome sets) or genome size, seen from an increase in size or dimension of the polyploids compared with the diploids (or lower ploids). To identify nucleotypic effects, confounding effects of hybridity (observed in allopolyploids), postpolyploidization processes or environmental effects need to be considered. We morphometrically analysed five ploidy cytotypes of the sexual–apomictic species *Potentilla puberula* cultivated *ex situ* under the same experimental conditions. Sexuals are mainly tetraploid, while higher ploidy (penta- to octoploidy) is typically associated with the expression of apomixis. The cytotypes likely arose via autopolyploidization although historic involvement of another species in the origin of apomicts cannot be fully ruled out, suggested by a slight molecular differentiation among reproductive modes. We (i) revisited molecular differentiation using amplified fragment length polymorphisms and performed a morphometric analysis to test (ii) if cytotypes are morphologically differentiated from each other and (iii) if the size of individuals is related to their ploidy. Weak molecular differentiation of sexual versus apomictic individuals was confirmed. Cytotypes and reproductive modes were also morphologically poorly differentiated from each other, i.e. apomicts largely resampled the variation of the sexuals and did not exhibit a unique morphology. Overall size of individuals increased moderately but significantly with ploidy (ca. 14 % in the comparison of octo- with tetraploids). The results support an autopolyploid origin of the *P. puberula* apomicts and suggest a nucleotypic effect on overall plant size. We discuss taxonomic consequences of the results in the context of data on reproductive relationships among cytotypes and their ecological preferences and evolutionary origin, and conclude that cytotypes are best treated as intraspecific variants within a single species.

## Teaser

Although autopolyploidy is a well-established phenomenon, there are astonishingly few detailed quantitative studies on associated morphological variation, a traditional and indispensable aspect of systematic work. We morphometrically analysed five ploidy cytotypes in the rosaceous species *Potentilla puberula* and found them insignificantly differentiated from each other, supporting the notion that autopolyploids usually resample the morphospace of their ancestors. However, we observed nucleotypic effects seen from an increase in the overall size of individuals with ploidy. We critically discuss results in the context of postpolyploidization effects and the modifying influence of the environment, factors known to modify the morphology of cytotypes.

## Introduction

The number of chromosome sets in the cell nucleus, referred to as ploidy level (Winkler 1908), is an evolutionary highly important karyological feature which potentially affects the development, physiology, reproductive system or anatomy and morphology of an organism (Otto and Whitton 2000; Wendel 2000; Comai 2005). Polyploids, which carry more than two chromosome sets per nucleus, thus often differ from their diploid relatives in functional and structural traits. In case of autopolyploids, which arose from within a species (Kihara and Ono 1926), differences among the di- and polyploids primarily can be attributed to so-called nucleotypic effects, i.e. to effects from the number of monoploid genomes *per se* or to the DNA content of nuclei independently of the informational content (Bennett 1971, 1987; Levin 2002). Nucleotypic effects on the morphology and the anatomy of plants are observed on different organizational levels (Ramsey and Schemske 2002). Cell size increases in tendency with ploidy level or genome size (Bennett 1987; Beaulieu *et al.* 2008; Balao *et al.* 2011; Doyle and Coate 2019). On the tissue level, quantitative changes like the density of stomata or hairs were reported (e.g. Sosa *et al.* 2012; Sosa and Dematteis 2014; Chansler *et al.* 2016), while on the organismic level polyploidization can be associated with an increase in the organ size (like flowers or leaves) or in whole individuals (e.g. Sosa *et al.* 2012; Hodálová *et al.* 2015).

Nucleotypic effects can easily be confound by other evolutionary processes or phenomena. Apart from the effects of hybridity, which occur in allopolyploids (i.e. polyploid hybrids) (Kihara and Ono 1926; Ramsey and Schemske 1998, 2002), ecological differentiation and postpolyploidization processes can mask nucleotypic effects on plant traits. Polyploids are often ecologically differentiated from their di- or lower ploidy ancestors (Bayer *et al.* 1991; Felber-Girard *et al.* 1996; Baack 2004; Sonnleitner *et al.* 2010) leading to the difficulty to separate environmental effects on plant traits from the nucleotypic effects. A strategy applied to minimize environmental effects is cultivation and study of the cytotypes under identical conditions (e.g. Mráz *et al.* 2011). Effects of postpolyploidization processes (Levin 1983) such as temporal diversification of cytotypes could be identified by increased genetic differentiation (e.g. Hodálová *et al.* 2015).

Polyploidization is often associated with changes in the reproductive mode of polyploids compared with their diploid ancestors such as the breakdown of self-incompatibility systems (Barrett 1988) or the evolution of so-called gametophytic apomixis (Carman 1997). Gametophytic apomixis refers to a mode of asexual formation of seeds common in the Asteraceae, Poaceae, Ranunculaceae and Rosaceae (Asker 1980; Asker and Jerling 1992). It is derived from sexual backgrounds (Van Dijk and Vijverberg 2005) and its origin is usually connected to a raise in ploidy level in the apomictic forms compared with the sexual ancestor(s). In most cases, the ancestral sexuals are diploid, thus giving rise to sexual diploid–apomictic polyploid contrasts (e.g. Bayer 1997; Hojsgaard *et al.* 2008; Cosendai *et al.* 2011; Paule *et al.* 2011; Uhrinová *et al.* 2017), but reproductive differentiation at the polyploid level also exists as exemplified in some species of the genus *Hieracium* from Asteraceae (Rotreklová *et al.* 2002) or in the rosaceous genus *Potentilla* (Czapik 1961; Smith 1963, 1971; Dobeš *et al.* 2013*b*, Paule *et al.* 2015).

Polyploidy in gametophytic apomicts (for convenience we refer henceforward to gametophytic apomixis as apomixis) is one of the two commonly distinguished main types: the majority of apomicts are of allopolyploid origin (e.g. Böcher 1951; Asker 1970*a*, 1970*b*, Campbell and Wright 1996; Bayer 1997; Hörandl and Gutermann 1999; Paule *et al.* 2012), whereas autopolyploid apomicts appear to be rarer. In most cases, autopolyploid apomicts are derived from diploids (e.g. in *Paspalum* L.: Hojsgaard *et al.* 2008; *Ranunculus* L.: Cosendai *et al.* 2011; *Townsendia* Hook.: Thompson and Whitton 2006; *Sorbus* L.: Lepší *et al.* 2015; Feulner *et al.* 2017), but derivation of (high) autopolyploids from tetraploids is also known (Mráz *et al.* 2008; Dobeš *et al.* 2013*b*).

A comparatively well-studied example of sexual–apomictic differentiation at the polyploid level is *Potentilla puberula* Krašan (= *Potentilla pusilla* Host: Soják 2010). During the last decade the species has been established as an evolutionary model to study the consequences of reproductive mode differentiation particularly from the spatial and ecological point of view (Hülber *et al.* 2013; Dobeš *et al.* 2015; 2017*a*; Alonso-Marcos *et al.* 2019). *Potentilla puberula* exhibits sexual–apomictic differentiation into five ploidy cytotypes: tetraploids being almost exclusively sexual and penta- to octoploids reproducing via apomixis (Prohaska 2013; Dobeš *et al.* 2013b, 2017*b*). DNA–molecular relationships among the five cytotypes, established using amplified fragment length polymorphism (AFLP) fingerprinting and cpDNA sequencing, suggested that both new apomictic and sexual genotypes arise within the species (Nardi *et al.* 2018). Interestingly, at least one apomictic parent is required for the origin of a novel apomictic genotype, compatible with the idea of a reproductive transfer of the apomictic trait. The study also uncovered existence of sexually reproducing hexa- and pentaploids, but they were solely derived from the sexual tetraploids, and at very low frequencies. Autopolyploidy of the apomictic cytotypes was also supported by the fact that no traces of other studied congeners were recovered in their genomes. The overall evolutionary relationships among the sexuals and apomicts, nevertheless, did not become fully clear: The first coordinate of a principle component analyses based on 370 polymorphic AFLP fragments (scored for 726 individuals) separated the sexuals (mainly tetraploids) from the apomicts. However, the differentiation was probably mainly due to the three apomixis-linked AFLP fragments and largely disappeared when these were removed from the analysis. The pattern was explained either by current directed gene flow from the sexuals to the apomicts followed by selection for genotypes possessing the three fragments or by historic introgression of the apomicts by an extinct or unsampled species.

*Potentilla puberula* belongs to the *Potentilla verna* aggregate, a complex containing at least five additional species (Ehrendorfer 1973), from which it is distinguished (in the alpine regions of Central Europe) by the largely constant possession of sparse stellate hairs and glandular pedicels (Dobeš 1999). *Potentilla puberula* is a morphologically highly diverse species, which complicates its distinction. Wolf (1908) distinguished, various varieties and taxonomic forms and particularly pointed at the high variability in characters describing the indumentum, the shape of basal leaves and the flower (Wolf 1903). However, the relationship between morphological variation and variability in ploidy or reproductive mode has not been studied yet. In addition, apomixis stabilizes (geno- and) morphotypes via clonal propagation potentially leading to prevalence of a limited number of more or less identical forms seen in discontinuities in the morphological variation of a species, a peculiarity also attributed to *P. puberula* (e.g. Wolf 1903, p. 46).

In the following study, we investigated the morphological relationships among the sexual tetraploid and the four apomictic high-ploidy cytotypes known for *P. puberula*. We were particularly interested in whether (i) the apomictic cytotypes are morphologically differentiated from each other and (ii) the apomicts resample the qualitative morphological variation of the sexuals, expected from their supposed autopolyploid origin. We further (iii), only considering the metric variables, test for effects of the number of monoploid genomes (i.e. nucleotypic effects) on overall plant size. We revisited the DNA–molecular differentiation of apomicts from the sexuals as has been observed by Nardi *et al.* (2018) in order to verify the relationships among cytotypes and to get additional evidence on the reproductive mode of individuals based on the presence and absence of apomixis-linked markers. The differentiation allowed distinguishing between sexual polyploids solely derived from the common sexual tetraploids and apomictic polyploids which received a genetic contribution from at least one apomictic parent, respectively.

## Materials and Methods

### Plant material

We performed the study on material collected in Eastern Tyrol, Austria, an area in which hybridization of *P. puberula* with other members of the *P. verna* aggregate can be excluded due to the absence of closely related congeners (Dobeš 1999; Polatschek 2000). Ninety-six individuals, representing all five known cytotypes, of *P. puberula* from 22 populations and cultivated in the experimental garden of the Institute of Pharmacognosy of the University in Vienna (48°13′56″N/16°21′37″E) were collected *ex situ* in spring 2013. Field collection was conducted in 2010 and all individuals were approximately of the same age when morphometrically analysed. Plants were grown in plastic pots (ø 14 cm) using a substrate composed of six parts ground soil, two parts of bark humus (Rindenhumus, Kranzinger, Straßwalchen, Austria) and two parts of quartz sand. We prepared herbarium vouchers from ground leaves and inflorescences using soft tissue and silica gel-dried young healthy leaves for the DNA extraction. Vouchers are deposited in the herbarium of the Natural History Museum in Vienna (W). Ploidy information of individuals was taken from Dobeš *et al.* (2013*b*), data on reproductive mode obtained using the flow cytometric seed screen (FCSS) (Matzk *et al.* 2000; Dobeš *et al.* 2013*a*) from Dobeš *et al.* (2013*b*, 2017*b*) (Table 1). Additionally, we included one pentaploid and one hexaploid individual (from population 6 Obermauern) reproducing sexually according to the FCSS, a sample frequency which approximately reflects the natural relative abundance of these rare sexual high ploidy cytotypes (Nardi *et al.* 2018).

**Table 1.** Characterization of the 96 individuals of *Potentilla puberula* from Eastern Tyrol studied by means of morphological and DNA-molecular (AFLP) variation. ‘Ploidy’, provided as the number of monoploid genomes (x), was derived from Dobeš *et al.* (2013*b*). We gathered data on reproductive mode (‘Apo’ apomictic and ‘Sex’ sexual) determined using the FCSS (Matzk *et al.* 2000) from Dobeš *et al.* (2013*b*) ‘Milosevic’ and from Dobeš *et al.* (2017*b*) ‘Fenko’. We further accepted the perfect link between reproductive mode and the occurrence of apomixis-linked AFLP fragments as a criterion to assign reproductive mode to individuals not screened by FCSS (‘AFLP phenotype’). The last column ‘Reproductive mode accepted’ summarizes the evidence on reproductive mode. Individuals for which we obtained the same results are joined in one line. ^a^‘Apo’ refers in this column to the ability of individuals to from seeds apomictically although apomixis may be facultative.

Population	Individual	Ploidy	AFLP phenotype	Milosevic	Fenko	Reproductive mode accepted^a^
1 Gonzach 46.87578°N/12.66265°E	4	5x	Apo	Apo		Apo
	15	6x	Apo			Apo
	28	6x	Apo	Apo		Apo
2 Unterleibnig 46.90337°N/12.63542°E	10	5x	Apo			Apo
3 Außer Klaunzer-Berg 46.97385°N/12.55678°E	43	5x	Apo			Apo
	38	7x	Apo			Apo
	45	7x	Apo	Apo		Apo
4 Oberpeischlach 46.93583°N/12.59405°E	18	5x	Apo			Apo
	28	7x	Apo			Apo
5 Rabenstein 47.00903°N/12.46575°E	47	4x	Sex			Sex
	24	5x	Apo			Apo
6 Obermauern 47.00472°N/12.43544°E	12	4x	Sex			Sex
	23, 27, 35	4x	Sex			Sex
	3	5x	Sex	Sex		Sex
	5	6x	Sex			Sex
	9	6x	Sex	Sex		Sex
7 Hainfels 46.75068°N/12.43715°E	3	4x	Sex			Sex
	20	4x	Sex	Sex		Sex
	49	5x	Apo			Apo
	32	7x	Apo			Apo
	43	7x	Apo	Apo		Apo
8 Bobojach 47.017°N/12.40368°E	16	4	Sex			Sex
9 Raut 46.78112°N/12.57448°E	5	4	Sex		Sex	Sex
	16, 47	4	Sex		Sex	Sex
	31	4	Sex	Sex	Sex	Sex
	12	5	Apo		Apo	Apo
	37	5	Apo	Apo	Apo	Apo
10 Zabernig 47.00467°N/12.5192°E	1, 8	4	Sex		Sex	Sex
	17, 22	4	Sex	Sex	Sex	Sex
	3, 33	5	Apo		mixed	Apo
	28	7	Apo		mixed	Apo
	26, 32	7	Apo		Apo	Apo
11 Kosten 46.78628°N/12.60243°E	32	7	Apo			Apo
	9	8	Apo	Apo		Apo
	2, 43	8	Apo			Apo
12 Hopfgarten 46.92607°N/12.52558°E	1	7	Apo			Apo
	45	7	Apo			Apo
13 Groder 47.01883°N/12.33275°E	15, 30	4	Sex	Sex		Sex
	20	4	Sex			Sex
	33	4	Sex		Sex	Sex
	5	5	Apo		Apo	Apo
	17	5	Apo	Apo	Apo	Apo
	16	6	Apo			Apo
14 Erlbach 46.74653°N/12.36964°E	12	5	Apo		Apo	Apo
	20	5	Apo		mixed	Apo
	6	7	Apo	mixed	Apo	Apo
	23, 32	7	Apo		Apo	Apo
	17	8	Apo	Apo		Apo
	19	8	Apo		Apo	Apo
	25	8	Apo	mixed		Apo
15 Lana 46.98575°N/12.6319°E	6, 37	5	Apo		Apo	Apo
	17	6	Apo			Apo
	33	6	Apo	Apo	mixed	Apo
	41	6	Apo		Apo	Apo
	46	6	Apo	Apo	Apo	Apo
	2	8	Apo	Apo		Apo
17 Stein 47.02757°N/12.52672°E	5, 30	5	Apo		Apo	Apo
	28	7	Apo		Apo	Apo
	29	7	Apo	mixed	Apo	Apo
	49	7	Apo	Apo	Apo	Apo
	15, 16	8	Apo		Apo	Apo
18 Innervillgraten 46.81183°N/12.36085°E	29	5	Apo	Apo	Apo	Apo
	1, 5	6	Apo		Apo	Apo
	32	6	Apo		Apo	Apo
	37	7	Apo		mixed	Apo
	27, 44	8	Apo		Apo	Apo
20 Dorfmäder 47.02528°N/12.36367°E	7	4	Sex	Sex		Sex
	13	4	Sex			Sex
	25	4	Sex			Sex
	43	4	Sex			Sex
21 Moaalm 47.03358°N/12.62811°E	16	5	Apo	Apo		Apo
	14	6	Apo	Apo		Apo
	21	6	Apo			Apo
	8	7	Apo			Apo
22 Katalalm 47.05761°N/12.48822°E	16	5	Apo			Apo
38 Obergaimberg 46.84612°N/12.78215°E	13, 21	7	Apo			Apo
49 Ratzell 46.92555°N/12.53908°E	6	5	Apo		Apo	Apo
	17	5	Apo		Apo	Apo
	13	6	Apo		Apo	Apo
	28	6	Apo		Apo	Apo

### Amplified fragment length polymorphisms

Amplified fragment length polymorphisms were analysed applying the protocol established by Vos *et al.* (1995) with few modifications as described in Paule *et al.* (2011) using EcoRI-AGG [NED]/MseI-CTC, EcoRI-AAC [6-FAM]/MseI-CTT, EcoRI-AGC [VIC]/MseI-CTG as three selective primer pairs. Differentially fluorescence labelled PCR products and GS600 LIZ size standard (Applied Biosystems, USA) were multiplexed and the fragments were separated on a 3730 DNA Analyser (Applied Biosystems, USA). A total of 96 samples and 8 repeat samples were analysed. Raw data were visualized and scored using GeneMarker version 1.90 (SoftGenetics, USA) and exported as a presence/absence matrix.

For the AFLP analyses, the following measures were computed using the R-script AFLPdat (Ehrich 2006) for the whole dataset and the particular cytotypes: total number of fragments, proportion of polymorphic fragments and number of private fragments. Assignment to sexual and apomictic lineages was carried out on the basis of the apomixis-linked AFLP fragments identified previously (Nardi *et al.* 2018*b*) combined with the data from the FCSS (see above, Table 1).

To test whether sexual and apomictic individuals can be discriminated based on the AFLP phenotypes (i.e. the diagnostic value of apomixis-linked fragments), a discriminant analysis of principal components (DAPC) (Jombart *et al.* 2010) was applied, using the *adegenet* package (Jombart 2008) in R (R-Development-Core-Team 2011). Reproductive mode of individuals inferred from the FCSS was then plotted onto the discriminant component.

### Morphometric analysis

Thirty-nine metric, three ordinal and two nominal morphometric variables (Table 2) were scored. The variables describe plant architecture, the shape and the size of generative and vegetative organs, and indumenta, features representing traits which were identified as being useful to distinguish *Potentilla* species and in particular members of the *Potentilla verna* aggregate (Wolf 1908; Dobeš 1999). Morphometric measurements were performed on plane, dried specimens. Basal leaves including stipules were prepared separately. Morphological structures were measured and analysed using a ruler or ocular micrometers installed on a Nikon SZU binocular (Nikon, Japan) and Reichert Biovar light microscope (Reichert, Austria), both equipped with epi-illumination.

**Table 2.** Definition of 44 morphological variables screened for *Potentilla puberula*. The scale of variables (39 metric ‘m’, 3 ordinal ‘o’, and 2 nominal ‘n’ ones) is provided and the expression of the character states used for ordinal and nominal characters given in brackets. The number preceding the name of the variables is that we refer to in the main text.

*Basal leaves*
1 number of leaflets: m; 2 length of central leaflet: m; 3 width of central leaflet: m; 4 position of maximum width of central leaflet measured from its basis: m; 5 length of uppermost lateral tooth of central leaflet: m; 6 width of uppermost lateral tooth of central leaflet: m; 7 number of teeth of central leaflet: m; 8 position of the notch formed by the lowermost lateral tooth of central leaflet measured from its basis: m; 9 position of the notch formed by the uppermost lateral tooth of central leaflet measured from its basis: m; 10 color of teeth tips: n (0 = green, 1 = pale red, 2 = intensive red); 11 length of petiole: m; 12 length of petiole plus leaf ground (the adnate region of the stipule): m; 13 length of stipules: m; 14 width of stipules: m; 15 length of the central ray of stellate hairs: m; 16 number of lateral rays of stellate hairs: m; 17 petiole with glands: n (0 = Yes, 1 = No); 18 off-axis angle of guard hairs of the petiole: o (1 = 90°–60°; 2 = 60°–30°; 3 = 30°–0°); 19 maximal length of guard hairs of the petiole: m
*Flowers*
20 diameter of flowers (as defined by the petals): m; 21 diameter of the discus: m; 22 length of petals: m; 23 width of petals: m; 24 position of maximum width of petals measured from its basis: m; 25 depth of the notch of the petals: m; 26 length of anthers: m; 27 width of anthers: m; 28 length of peduncle: m; 29 length of sepals: m; 30 width of sepals: m; 31 length of outer sepals: m; 32 width of outer sepals: m; 33 off-axis angle of guard hairs of the peduncle: o (1 = 90°–60°; 2 = 60°–30°; 3 = 30°–0°); 34 maximal length of guard hairs of the peduncle: m
*Flowering shoots*
35 number of flowers per inflorescence: m; 36 number of cauline leaves: m; 37 number of leaflets of lowermost cauline leaf: m; 38 length of central leaflet of lowermost cauline leaf: m; 39 width of central leaflet of lowermost cauline leaf: m; 40 number of teeth of central leaflet of lowermost cauline leaf: m; 41 length of central axis of the inflorescence: m; 42 total length of the flowering shoot: m; 43 times of branching of inflorescence: o; 44 distance of lowermost branch from the basis of the flowering shoot: m

Statistical analyses were carried out using R (R Development Core Team 2011). The correlation coefficient among all pairwise combinations of variables was computed using the *cor* function in order to detect undesirable high correlations (*r* ≥ 0.95). We used Pearson’s correlation coefficients (*r*) for metric variables and Spearman’s correlation coefficient (*rho*) when the ordinal variables were included. After exclusion of highly correlated variables, we run a principle component analysis (PCA) using the eigen function and visually explored the relation of cytotypes and reproductive modes. A discriminant analysis (DA) was carried out on the metric variables to identify taxonomically useful characters using the *lda* function from the *MASS* library. The prior probabilities of class membership were defined as group proportions. Variables were scaled to zero mean and unit variance to balance the effect of different value ranges. To obtain better separation of cytotypes, we also run DAs for all 10 possible pairs of cytotypes. For these analyses, we created boxplots for variables that show high correlations with the linear discriminants. To explore whether plant size influences the separation of cytotypes, we run two DAs only using the metric variables: one analysis was run on the original data and the second was run after normalization of data, which means dividing the values observed for the various variables for an individual by the sum of these values. This normalization reduces variation to differences in shape. Finally, an effect of the number of monoploid genomes on the size of individuals was tested in normalizing metric variables representing size (2, 3, 5, 6, 11–15, 19–21, 23, 24, 27–33, 36, 40, 41, 43, 44; Table 2) through division by the column sums (the sum of all values observed for a variable in the data). Thereby we removed effects from absolute size differences among variables (which otherwise would result in a higher weight of larger characters compared with smaller ones), but kept the relative size relations among individuals. These relative measures of size were then summed up over all variables for each individual and regressed against ploidy of individuals using the *lm* function. Since the correlation was significant, we also run the regression on the single variables to identify their respective role.

## Results

### Amplified fragment length polymorphisms

Three AFLP primer combinations resulted in 129 clearly scorable fragments in total (43–67 per sample) sized from 93 to 569 bp; of which, 92.24 % were polymorphic. The repeatability of the data ranged between 97.75 % and 100 %. The number of fragments per individual was slightly higher in higher ploids than tetraploids, including 1–7 private fragments per particular ploidy level.

**Figure 1.**
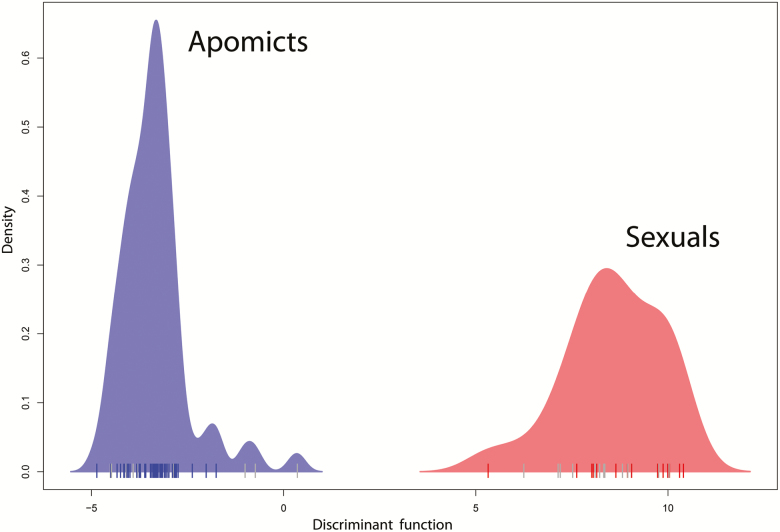
Discriminant analysis of principal components (DAPC) based on AFLP phenotypes with a priori determined groups carrying (‘apomicts’) and missing (‘sexuals’) apomixis-linked fragments, respectively. The vertical bars above the *x*-axis mark the position of particular individuals. Blue and red bars denote individuals for which FCSS suggested apomictic and sexual reproduction, respectively. Grey bars symbolize individuals not screened for reproductive mode.

In our dataset, two out of the three apomixis-linked AFLP fragments recognized by Nardi *et al.* (2018) were recovered, but shifted in length by 3 bp (fragments 216 bp and 282 bp, selective primer combination EcoRI-AAC/MseI-CTT) most probably due to methodological issues (different enzyme and sequencer manufacturer). All individuals (exclusively penta- to octoploids) able to reproduce apomictically based on FCSS (Table 1) carried both apomixis-linked fragments while individuals (all tetraploids, one penta-6-03, und two hexaploids 6-05, 6–09) missing these fragments formed seeds via regular sexuality (Table 1). Individuals carrying and missing the apomixis-linked fragments were clearly separated by the DAPC analysis retaining 40 PCs with a proportion of conserved variance of 0.914 explained by the discriminant function (Fig. 1). Based on the perfect link between the fragments and reproductive mode, we considered all individuals carrying the apomixis-linked fragments ‘apomictic’ and those missing the fragments ‘sexual’ (Table 1, ‘reproductive mode accepted’).

### Morphometry

**Figure 2.**
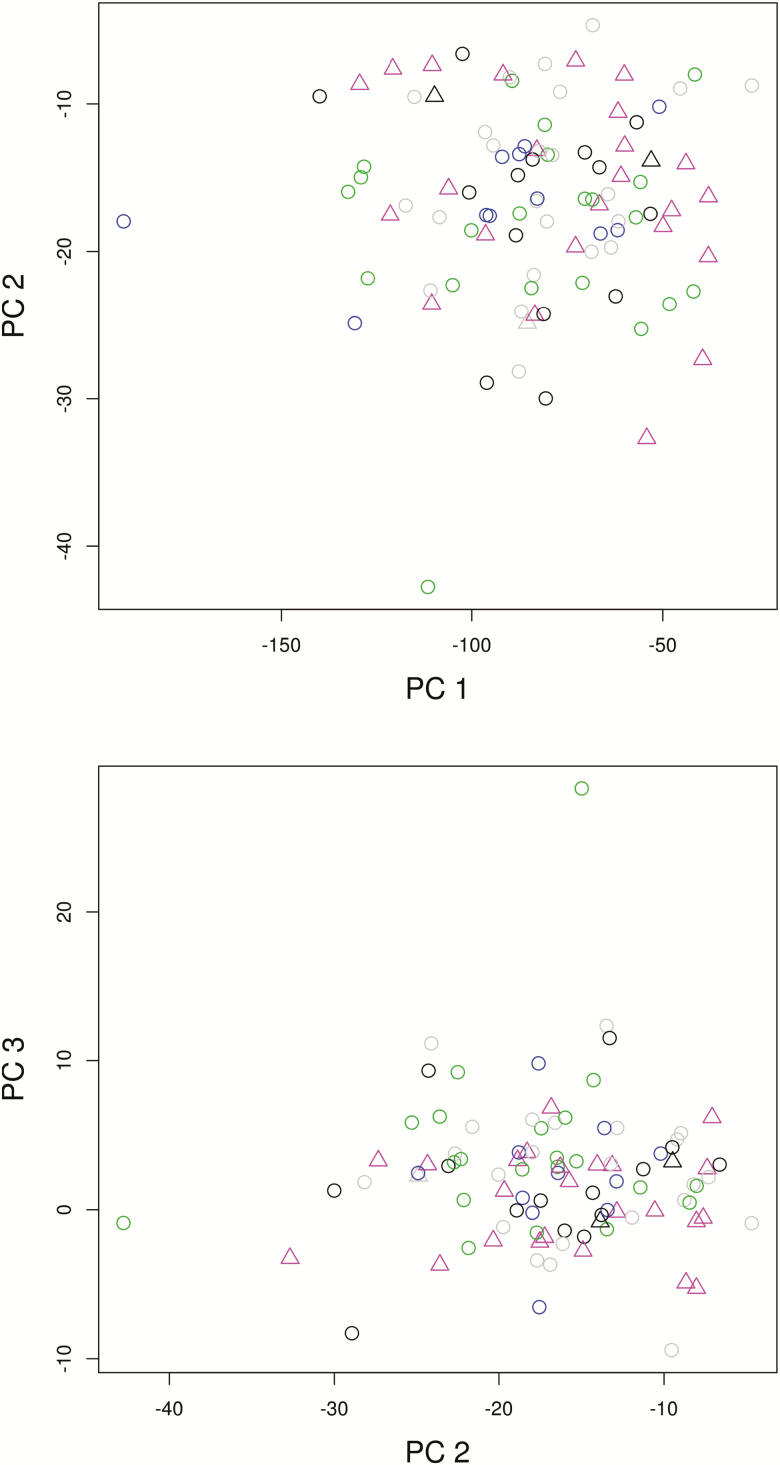
Principal component analysis (PCA) of morphological characters scored for 96 individuals of *Potentilla puberula* labelled by ploidy (pink, tetraploid; grey, pentaploid; black, hexaploid; green, heptaploid; blue, octoploid) and reproductive mode (triangles, sexual; circles, apomictic). First three principal components explained 95.50 % of the variation in the data.

All morphometric characters were variable (**see****Supporting Information—File S1**). Five metric variables were omitted from further analyses because of high correlation with another character (Pearson correlation coefficient (*r*) > 0.95). We subjectively kept the characters which we considered more intuitive in describing the morphology of the species in a taxonomic context: e.g. character 2 ‘length of central leaflet’ instead of 8 ‘position of the notch formed by the lowermost lateral tooth of central leaflet measured from its basis’ and 11 ‘length of petiole’, character 41 instead of 44, and 3 instead of 4 and 9 (cf. Table 2). The first three components of the PCA explained 87.99, 4.85 and 2.66 % (95.50 % in total) of the variation in the data. There was no obvious differentiation or grouping of individuals neither by ploidy nor by reproductive mode (Fig. 2).

**Figure 3. Variation:**
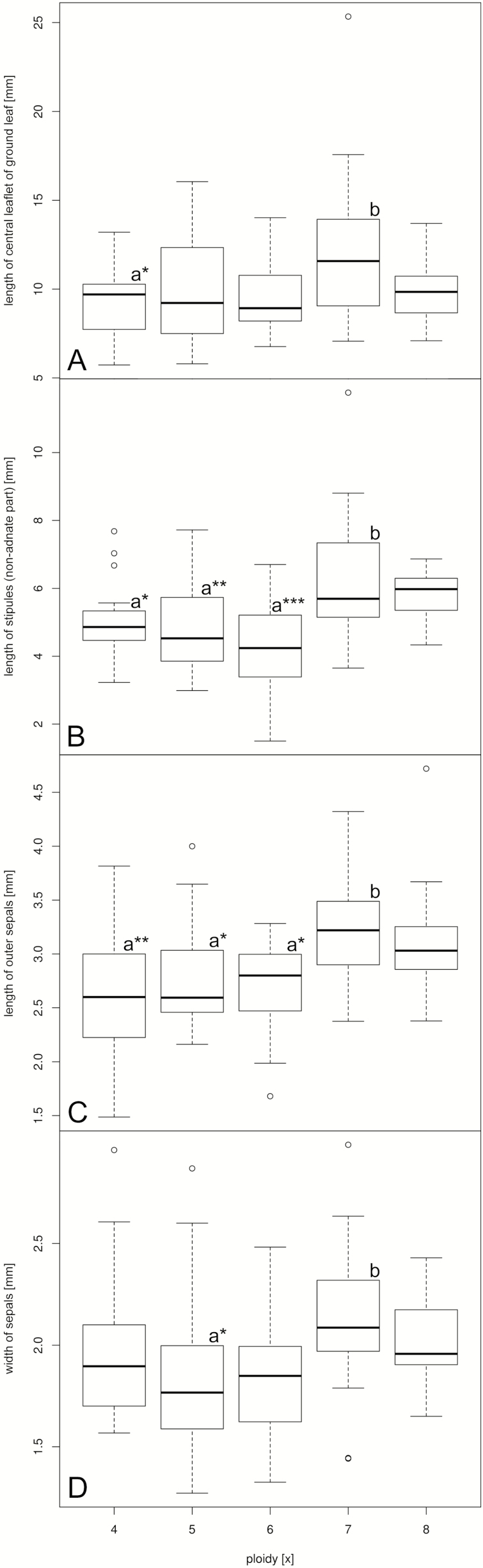
in four morphometric characters which showed significant differences among at least one pair of cytotypes (indicated by lower case letters). ***, ** and * refer to statistic significance at the *P* < 0.001, < 0.01 and < 0.05 significance level, respectively (pairwise *t*-test corrected for multiple comparisons).

The DA based on the 33 metric variables classified only 10.4, 8.3, 7.3, 8.3 and 2.1 % (36.5 % in total) of the tetra-, penta-, hexa-, hepta- and octoploid individuals, respectively, according to their ploidy (the prior assignment). Exclusion of size of individuals did deteriorate the overall classification probability of cytotypes to the a priori defined groups (11.5, 8.3, 6.3, 8.3, 2.1 % for the five cytotypes, 36.5 % in total). Using reproductive mode as grouping criterion, 64.6 % of the apomicts and 16.7 % of the sexuals were assigned to their own group. Only 4 out of the 12 characters selected based on their relatively high correlations with the first four linear discriminants (Table 3) differed significantly for at least one pair in the DAs run for the 10 pairs of cytotypes (Fig. 3).

**Table 3.** Total canonical structure showing the correlation of the measured characters (Table 2) with the first four canonical axis. Highest values are given in bold.

Character	can1	can2	can3	can4
2	**0.3905**	−0.1223	0.0069	−0.2834
3	0.2810	0.1092	−0.0318	−0.3405
4	0.0778	0.2608	0.0893	**−0.2203**
5	0.3194	0.0813	−0.0410	−0.2680
6	0.2549	0.1787	−0.1429	**−0.3649**
7	−0.1646	−0.2080	−0.3060	0.0229
12	0.1046	0.0195	−0.1114	−0.1775
13	**0.5135**	0.1017	0.2291	−0.2237
14	0.1733	0.2771	0.0466	−0.3183
15	0.3017	0.0259	0.0548	−0.0714
16	−0.1227	0.0304	**0.6135**	−0.0621
19	0.2586	0.0151	−0.1239	−0.0432
20	0.0861	0.2469	0.2061	−0.2128
21	0.1931	0.0439	−0.3332	−0.1908
22	0.0183	0.2267	0.1579	−0.2506
23	−0.2138	0.2454	0.2730	−0.3126
25	−0.0014	0.0893	−0.0836	0.0837
26	0.0043	0.2172	0.2100	−0.2894
27	0.1190	**0.2836**	0.2011	−0.2261
28	0.3143	−0.0436	−0.2135	−0.0818
29	0.2842	**0.3554**	0.0754	−0.1202
30	0.2338	0.1320	0.2737	**−0.3244**
31	**0.5257**	0.1200	−0.0471	−0.1875
32	0.0470	0.2392	0.0692	−0.1365
34	0.1237	−0.0235	−0.0830	−0.2429
35	0.1192	−0.1073	−0.1237	0.0511
36	0.0771	−0.0470	−0.1694	0.0982
37	−0.2711	0.1272	**−0.4165**	−0.0595
38	0.1570	0.2048	−0.0405	0.2880
39	0.1174	**0.3388**	−0.1774	0.3154
40	−0.0723	0.1411	**−0.3786**	0.2931
41	0.1495	0.1319	−0.1879	0.0288
42	0.1518	0.1196	−0.1466	0.0314

**Figure 4. Regression:**
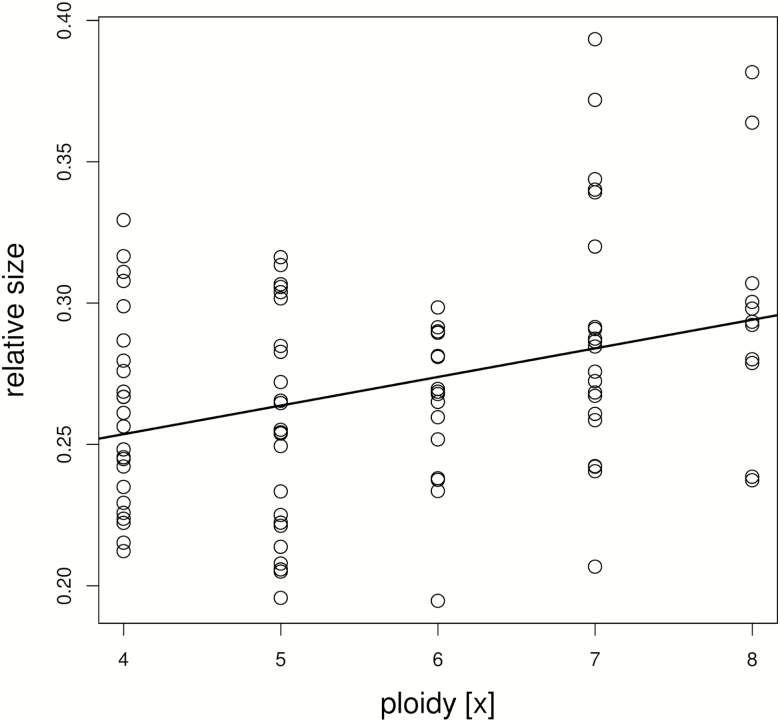
of overall relative size of individuals against their ploidy. The positive relation suggests a slight but significant effect of number of monoploid genomes (i.e. a nucleotypic effect) on plant size (linear regression, *F* = 12.03, *P* = 0.00079).

Overall relative size of individuals was significantly positively correlated with the ploidy, indicating an effect of the number of monoploid genomes (linear regression, *F* = 12.03, *P* = 0.00079; Fig. 4). Eleven characters (2, 3, 5, 6, 13, 14, 19, 21, 28, 29, 31; Table 2) were significantly (positively) correlated with ploidy in the single analyses (*F* = 17.98 to 4.14, *P* ≤ 0.045). The characters describe size of the central leaflet of the basal leaves, the sepals and the outer sepals, the diameter of the flower discus, and the length of the peduncle and guard hairs.

## Discussion

We studied the molecular relationships among five cytotypes of *P. puberula*. In accordance with Nardi *et al.* (2018) individuals were separated by reproductive mode but not according to ploidy (due to the occurrence of penta- and hexaploid sexuals). The pattern was explained by Nardi *et al.* (2018) by occasional derivation of sexually reproducing hexa- and pentaploids from the sexual tetraploids on the one hand, and obligatory involvement of at least one apomictic parent in the origin of new apomicts on the other hand. In contrast, differentiation among cytotypes as well as reproductive modes (sexual versus apomictic) was very poor based on morphometric characters. The data indicated that the apomicts largely resample the morphological variation of the sexuals and that the single apomictic cytotypes do not present unique morphologies, in accordance with an autopolyploid origin.

Although known as a phenomenon since long (Kihara and Ono 1926), autopolyploidy only quite recently (but see for instance Müntzing 1936 for early interests in the topic) came into the focus of plant systematists (Soltis *et al.* 2007). While a wealth of data on the geographic distribution of cytotypes (e.g. Kay 1969; Nesom 1983; Soltis 1984; Krendl 1993; Dobeš and Vitek 2000; Lihová and Marhold 2003; Šmarda and Bureš 2006; Cosendai *et al.* 2011; Paule *et al.* 2017), their ecological preferences (e.g. Rothera and Davy 1986; Lumaret *et al.* 1987; Felber-Girard *et al.* 1996; Sonnleitner *et al.* 2010; Paule *et al.* 2018) or reproductive compatibility (Zohary and Nur 1959; Kay 1969; Levin 1975; Van Dijk *et al.* 1992; Hardy *et al.* 2001) has been gathered, there are astonishingly few detailed quantitative studies on their morphological differentiation, a traditional and almost indispensable aspect of systematic work (Stuessy 2009). The majority of studies on morphological differentiation of cytotypes in autopolyploid systems is largely observational and restricted to comparison of few characters with differences generally reported to be small (e.g. Mosquin 1967; Hunzinker *et al.* 1972; Nesom 1983; Soltis *et al.* 2007) or non-recognizable (e.g. Bayer 1991; Thompson *et al.* 2004; Jorgensen *et al.* 2008). Results from morphometric studies, for which usually more representative number of characters was screened, are non-unequivocal. Some studies found either relatively weak (Marhold 1999; Hodálová *et al.* 2007) or missing (Saukel and Länger 1990; Feulner *et al.* 2017) differentiation between diploids and the autopolyploid derivatives. Our results are in good accordance with these studies and support the notion that autopolyploids usually resample the morphospace of their parents (Soltis *et al.* 2007). For instance, a quite similar system exists with *Pilosella rhodopea* (Asteraceae) which shows infraspecific ploidy differentiation (di-, tri-, tetra- and pentaploid) as well as sexual–apomictic differentiation. Analogous to our results, the ploidy cytotypes did not show a clear pattern of morphological differentiation in a PCA based on 42 characters, suggesting an autopolyploid origin of the cytotypes, a conclusion also backed by nuclear ribosomal DNA sequence data (Šingliarová *et al.* 2011). In contrast, for other systems a much stronger effect of autopolyploidy on morphology (and anatomy) was claimed as discussed by Chansler *et al.* (2016) for the genera *Centaurea* and *Jacobea* (both Asteraceae), *Stemodia* (Plantaginaceae) and *Larrea* (Zygophyllaceae). However, this claim should be taken with care because in the cited cases, the comparatively clear separation of the cytotypes may have other causes then nucleotypic effects per se. For *Centaurea stoebe* s.l. an allopolyploid origin of the studied tetraploids was demonstrated (Mráz *et al.* 2011, 2012). In case of genus *Stemodia*, hard evidence for autopolyploidy in the species was not provided (Sosa *et al.* 2012; Sosa and Dematteis 2014). In *Jacobea vulgaris* the morphologically distinct octoploids were also genetically strongly differentiated from their tetraploid relatives and postpolyploidization processes or alternative scenarios of the origin of the octoploids were considered (Hodálová *et al.* 2015). Finally, morphological variation among three cytotypes of *Larrea tridentata* analysed by Laport and Ramsey (2015) on material collected in the wild was at least partly attributed by the authors to the ecological differentiation among cytotypes, their allopatric geographic distribution and possibly genetic divergence.

The observed poor morphological differentiation of the *P. puberula* ploidy cytotypes was in contrast to the clear separation of individuals by reproductive mode in the AFLP-based analysis (Fig. 1). The separation could be largely attributed to the recovery of two out of three apomixis-linked AFLP fragments described by Nardi *et al.* (2018). The authors speculated that the fragments are linked to a genomic region or regions which are functionally related to the expression of apomixis (see Ozias-Akins and Van Dijk 2007) and are selected for to make this reproductive mode functional.

The genetic basis of apomixis in *Potentilla* is largely unknown (Asker 1980). However, provided that the hypothesis proposed by Nardi *et al.* (2018) holds true, the evolution of the genomic region coding for apomixis may qualify as a postpolyploidization process. This idea is not unrealistic since gametophytic apomixis is a highly polyphyletic trait (Van Dijk and Vijverberg 2005) which evolved from sexual backgrounds involving polyploidization of the genome (Carman 1997; Hand and Koltunow 2014). Our morphometric results would be in accordance with this scenario, i.e. that the apomictic cytotypes arose within the species, followed by only little morphological and molecular postpolyploidization differentiation. Alternatively, the slight molecular differentiation may indicate involvement of past introgression in the origin of apomicts from an unknown species. However, hybridization commonly gives rise to intermediate morphotypes which differ from the parental ones (Vit *et al.* 2014; Christensen *et al.* 2014; Hajrudinovic *et al.* 2015) and genotypes exhibiting a proportion of markers from both parents (e.g. Uhrinová *et al.* 2017) or with a proportion of unique markers in case one parent was unsampled (e.g. Paule *et al.* 2011), situations not observed in *P. puberula*. Nevertheless, it must be noted that hybrids can morphologically more closely resemble their parents than expected, for instance, due to unequal genomic parental contributions, maternal inheritance or epigenetic effects (see discussion in Hodac *et al.* 2014).

On the basis of the canonical structure of the Das, we identified four characters which significantly differed for at least one pair of cytotypes (Fig. 3). All these pairs involved one high-ploidy cytotype (hepta- or octoploid), which consistently showed in average higher values for the character compared with the lower ploidy cytotypes. We therefore interpret the differences as nucleotypic effects, although a significant effect of size on the overall classification of cytotypes was not evident in the DAs since the classifications obtained from DAs run on the normalized compared with original data did not exacerbate.

We inferred a significant positive relation between size and ploidy both for overall size (Fig. 4) and several single characters of different plant organs (see Results). The correlation was not unexpected since an increase in the number of monoploid genomes is known to enlarge cell size (Müntzing 1936; Stebbins 1971) either via the sheer space required by the enlarged nuclei and its positive correlation with cell size or gene dosage effects (Bennett 1971; Levin 2002; Doyle and Coate 2019). The volume of tetraploid cells typically is about twice that of their diploid counterparts (Levin 2002). Plant size of autopolyploids thus could be expected to exceed the dimensions of their diploid counterparts, but size is counteracted by typically lower growth rates of polyploids compared with diploids (Müntzing 1936; Gottschalk 1976; Bennett 1987). In our study system, the effect of ploidy level on overall plant size was weak. In average, a duplication of the number of monoploid genomes (i.e. tetra- versus octoploidy) increased overall size by ca. 14 %.

It should be noted that the study has been performed on cytotypes cultivated under identical garden conditions, by which we aimed to minimize modificatory effects exerted by environmental conditions. The weak contrast in size among cytotypes may not be representative for natural populations since sexuals and apomicts show significant differentiation in their environmental preferences. Sexual tetraploids prefer primary habitats at drier, steeper, more south-oriented slopes, while apomicts mostly occur in human-made habitats with higher water availability (Alonso-Marcos *et al.* 2019). In the field, apomicts appear to be usually larger with more elongated axis and leaves—probably due to their preference for meadows—than sexual, which prefer more open and rocky sites (Ch. Dobeš, personal observation). Hence we may hypothesize that the difference in morphology among cytotypes under field versus high morphological similarity under experimental conditions may imply phenotypic plasticity of individuals (Sultan 2000) and lack of morphological adaptation of cytotypes to their respective preferred environments.

Our results have potential implications for the taxonomic treatment of *P. puberula*. Depending on the applied species concept (e.g. Hörandl 1998; Soltis *et al.* 2007), the different cytotypes may be treated as a separate species or considered intraspecific cytological variants. On the one hand, the sexual and apomictic cytotypes are ecologically differentiated in *P. puberula*. In addition, sexuals and apomicts spatially exclude each other (Hülber *et al.* 2013), a pattern explainable by reproductive suppression or competition (Levin 1975; Joshi and Moddy 1995). Reproductive suppression and ecological differentiation would be in favour of taxonomic differentiation of sexuals and apomicts. On the other hand, successful cross-fertilization among individuals of differing reproductive mode is quite easily possible (Dobeš *et al.* 2017b) and gives rise to novel cytotypes (Nardi *et al.* 2018). Apomictic cytotypes originated repeatedly, also involving contributions from the sexuals (Nardi *et al.* 2018). Hence, apomicts and sexuals are phylogenetically not separated or evolutionary independent from each other. Finally, high genetic and morphological similarity of cytotypes is in favour of a single species treatment when following a morphological species concept. On the basis of the total of these arguments, we argue that the cytotypes of *P. puberula* are taxonomically better treated within a single species.

### Sources of Funding

The project has been supported by the Austrian Science Fund (grant P27688 to C.D.) and internal funds of the Senckenberg Research Institute and Natural History Museum Frankfurt (J.P.).

### Contributions by the Authors

K.B. did the morphometric measurements and analysed and interpreted data. J.P. did the labwork, analysed and interpreted data. C.D. designed research, analysed data and wrote the manuscript.

### Conflict of Interest

None declared.

## Supporting Information

The following additional information is available in the online version of this article—

**File S1.** Descriptive statistics of 42 morphological characters screened for 96 individuals of *Potentilla puberula* representing five ploidy levels.

plz028_suppl_Supplementary-Material
