# Supplementary material for: The morphometrics of autopolyploidy: insignificant differentiation among sexual–apomictic cytotypes
Source: AoB Plants. 2019 Jun 4;11(3):plz028. doi: 10.1093/aobpla/plz028 (PMC6548344; doi:10.1093/aobpla/plz028)
Supplement: plz028_suppl_Supplementary-Material [file plz028_suppl_supplementary-material.pdf]

Supplementary Material 1 Descriptive statistics of -42 morphological characters screened for 96 individuals of *Potentilla puberula* representing five ploidy levels. Statistics is provided separately for normally distributed variables for which the mean and standard deviation (sd) is provided and non-normal distributed variables for which the median and the maximum and minimum value are given. For all variables the sample size (N) is given. Nominal characters are not presented.

ploidy		length of main leaflet	width of main leaflet	position of maximum width of central leaflet measured from its basis	position of the notch formed by the lowermost lateral tooth of central leaflet measured from its basis	position of the notch formed by the uppermost lateral tooth of central leaflet measured from its basis	length of petiole	length of petiole plus leaf ground (the adnate region of the stipule)	length of stipules	width of stipules	width of uppermost lateral tooth of central leaflet	length of uppermost lateral tooth of central leaflet	number of teeth of main leaflet	length of the central ray of stellate hairs	number of lateral rays of stellate hairs	maximal length of guard hairs of the petiole	diameter of flowers	length of petals	width of petals	position of maximum width of central leaflet measured from its basis	length of sepals	width of sepals	length of outer sepals	width of outer sepals	diameter of the discus	depth of the notch of the petals	length of anthers	width of anthers	number of flowers per inflorescence	maximal length of guard hairs of the peduncle	number of cauline leaves	number of teeth of main leaflet of lowermost cauline leaf	number of leaflets of lowermost cauline leaf	length of main leaflet of lowermost cauline leaf	width of main leaflet of lowermost cauline leaf	length of central axis of the inflorescence	total length of the flowering shoot	distance of lowermost branch from the basis of the flowering shoot	length of peduncle		number of leaflets of basal leaves	off-axis angle of guard hairs of the petiole	off-axis angle of guard hairs of the peduncle	times of branching of inflorescence
**tetraploid**	mean	9.11	5.09	6.67	5.48	8.14	14.18	18.41	4.90	0.66	0.96	1.04	8.67	0.82	4.75	1.33	13.78	5.31	5.03	3.50	3.41	2.00	2.64	1.24	1.78	0.34	0.62	0.38	2.88	0.83	1.04	2.85	5.25	5.09	2.46	46.94	54.81	27.37	12.13	median	5	2	2	1
	sd	2.01	1.11	1.48	2.00	1.76	5.38	5.94	1.08	0.20	0.23	0.30	1.63	0.23	3.04	0.45	2.02	1.05	0.92	0.76	0.60	0.38	0.58	0.43	0.28	0.18	0.07	0.07	1.12	0.22	0.62	0.37	1.41	1.25	0.86	19.98	20.88	12.26	4.45	minimum	5	1	1	0
	N	24																																						maximum	6	3	3	2
**pentaploid**	mean	9.78	4.93	7.15	5.17	8.46	14.50	18.65	4.78	0.54	0.92	1.05	9.58	0.86	2.42	1.46	12.49	4.66	3.96	3.10	3.24	1.82	2.77	1.12	1.88	0.35	0.55	0.34	3.33	0.84	1.29	2.86	6.05	5.46	2.64	50.90	58.44	28.79	15.04	median	6	2	2	1
	sd	2.85	1.50	2.43	2.16	2.52	5.48	6.19	1.18	0.21	0.22	0.56	2.06	0.47	2.17	0.48	2.03	0.99	0.89	0.72	0.50	0.37	0.48	0.25	0.22	0.30	0.13	0.07	1.46	0.25	0.75	0.64	1.99	1.70	0.77	13.87	15.71	11.37	4.86	minimum	5	2	1	0
	N	24																																						maximum	7	3	3	2
**hexaploid**	mean	9.54	5.31	6.90	4.88	8.21	16.02	20.24	4.26	0.64	1.06	1.11	10.25	0.79	1.31	1.44	12.82	4.98	4.50	3.34	3.27	1.83	2.70	1.18	2.03	0.36	0.58	0.35	3.19	0.90	1.25	3.46	6.38	4.79	2.51	52.58	59.14	28.84	15.01	median	5.5	2.5	2	1
	sd	2.13	0.91	1.86	2.25	2.16	6.04	6.94	1.35	0.29	0.20	0.21	3.24	0.22	1.70	0.54	2.22	1.16	1.05	0.89	0.59	0.30	0.46	0.28	0.21	0.15	0.13	0.09	0.91	0.26	0.86	0.78	1.66	1.40	0.50	16.61	16.22	12.76	5.29	minimum	5	1	0	1
	N	16																																						maximum	7	3	3	2
**heptaploid**	mean	12.03	5.98	9.12	7.27	10.06	15.32	20.57	6.26	0.73	1.14	1.37	8.67	1.00	3.14	1.59	13.74	5.22	4.42	3.52	3.59	2.13	3.24	1.22	2.00	0.32	0.61	0.39	3.29	0.94	1.19	2.63	4.95	5.13	2.38	52.15	60.03	29.02	17.02	median	6	2	2	1
	sd	4.15	1.54	3.56	3.03	3.20	5.69	6.74	1.90	0.26	0.41	0.58	2.29	0.24	3.21	0.44	2.59	1.23	1.31	0.93	0.49	0.37	0.48	0.26	0.21	0.13	0.09	0.07	1.27	0.28	0.60	0.76	1.81	2.18	0.92	19.52	21.18	9.99	8.67	minimum	5	1	1	0
	N	21																																						maximum	7	3	3	2
**octoploid**	mean	10.30	5.82	7.07	6.13	8.46	14.38	20.21	5.82	0.78	1.17	1.32	8.36	0.96	1.82	1.61	14.02	5.32	4.43	3.72	3.88	2.03	3.25	1.31	2.05	0.39	0.61	0.40	3.18	0.88	1.27	3.00	6.45	6.27	3.29	60.12	67.26	33.24	16.76	median	5	2	2	1
	sd	2.18	1.39	1.86	2.35	2.23	5.37	5.77	0.69	0.33	0.29	0.33	1.50	0.23	2.40	0.24	2.40	1.07	1.03	0.84	0.55	0.23	0.66	0.21	0.80	0.21	0.07	0.09	0.98	0.18	0.47	0.00	1.13	1.72	0.92	26.82	26.75	20.25	6.30	minimum	5	2	2	0
	N	11																																						maximum	7	3	2	2
